# The Effects of Structure and Oxidative Polymerization on Antioxidant Activity of Catechins and Polymers

**DOI:** 10.3390/foods12234207

**Published:** 2023-11-22

**Authors:** Wei Wang, Ting Le, Wei-Wei Wang, Jun-Feng Yin, He-Yuan Jiang

**Affiliations:** 1 Key Laboratory of Biology, Genetics and Breeding of Special Economic Animals and Plants, Ministry of Agriculture and Rural Affairs, Tea Research Institute, Chinese Academy of Agricultural Sciences, 9 Meiling South Road, Xihu District, Hangzhou 310008, China; ww1040491839@163.com (W.W.); 15968823529@163.com (T.L.);; 2 College of Horticulture, Fujian Agriculture and Forestry University, Cangshan District, Fuzhou 350002, China

**Keywords:** antioxidant activity, structure, oxidative dimerization, catechins, polymers

## Abstract

Polyphenols are key free radical scavengers in tea. This study screened the antioxidant active groups of catechins and dimers and analyzed the effects of the degree of oxidative polymerization and oxidative dimerization reaction on their antioxidant activities. ABTS^+·^ free radical scavenging activity, DPPH free radical scavenging activity, and total antioxidant capacity of catechins and polymers were systematically analyzed and compared in this study. Results manifested antioxidant activities of catechins were dominated by B-ring pyrogallol and 3-galloyl, but were not decided by geometrical isomerism. 3-galloyl had a stronger antioxidant activity than B-ring pyrogallol in catechins. The number, not the position, of the galloyl group was positively correlated with the antioxidant activities of theaflavins. Theasinensin A has more active groups than (−)-epigallocatechin gallate and theaflavin-3,3′-digallate, so it had a stronger antioxidant activity. Additionally, the higher the degree of oxidation polymerization, the weaker the antioxidant activities of the samples. The oxidative dimerization reaction hindered the antioxidant activities of the substrate–catechin mixture by reducing the number of active groups of the substrate and increasing the molecular structure size of the product. Overall, pyrogallol and galloyl groups were antioxidant active groups. The degree of oxidative polymerization and the oxidative dimerization reaction weakened the antioxidant activity.

## 1. Introduction

Antioxidation refers to resisting the peroxide state of living tissues induced by internal cellular metabolism or external stimuli. In the peroxide state, a balance between generating and eliminating reactive oxygen species (ROS) was broken. ROS content exceeding the scavenging capacity of the defense system will result in oxidative stress damage, such as DNA chain breakage, protein cross-linking, and lipid peroxidation, followed by the imbalance of intracellular metabolism.

One of the mechanisms leading to food spoilage, human diseases, and aging is the imbalance of intracellular metabolism [1,2]. Therefore, there is an urgent need for people to fight against oxidation, which made the search for auxiliary exogenous antioxidants recently become a research hotspot in biochemistry and medicine. As chemical antioxidants, such as butylated hydroxyanisole, may have potential carcinogenic risks in animal experiments [3], the following work is entrusted with a mission to focus on finding green and effective natural substances which have antioxidant properties that can act as substitutes for chemical synthetic antioxidants for safe, sustainable, and healthy development.

Owing to its potential health benefits, tea has long attracted much interest from researchers [4]. Its excellent antioxidant activity has been widely identified [5,6]. Fermentation is the key processing procedure to make black tea, in which the oxidative polymerization of catechins is very active. Oxidative polymerization of catechins is a crucial biochemical reaction to form the characteristics of black tea. When fresh tea leaves are fermented with polyphenol oxidase and peroxidase, catechins will be co-oxidated to form oxidized dimers (such as theaflavins (TFs) and theasinensins (TSs)) and polymers (such as thearubigins (TRs) and theabrownins (TBs)). It is reported that with the increased total phenolic content, the antioxidant activity of black tea is also enhanced [7]. Phenolic compounds were key free radical scavengers [8], such as catechins, TFs, TRs, and TBs [9,10]. In our previous experiments about the antioxidant activity of black tea processing samples, the decreased activity was found to accompany the progression of black tea fermentation. Meanwhile, the catechins content decreased gradually, the dimers’ content increased first and decreased later, and the polymer content increased gradually during the fermentation process [11]. Is the reduced antioxidant activity during black tea fermentation due to the lower activity of polymers compared to catechins and dimers or does the oxidative polymerization process reduce it? Additionally, different antioxidant activities of the samples could be found in different antioxidant methods (methods or conditions adopted in each protocol) [12]. It is difficult to generalize conclusions in some cases. Therefore, the antioxidant activities of catechins and their oxidized polymers were systematically studied using the same method in this research to investigate the effects of the degree of oxidation polymerization and the oxidative dimerization reaction on antioxidant activities.

**Figure 1:**
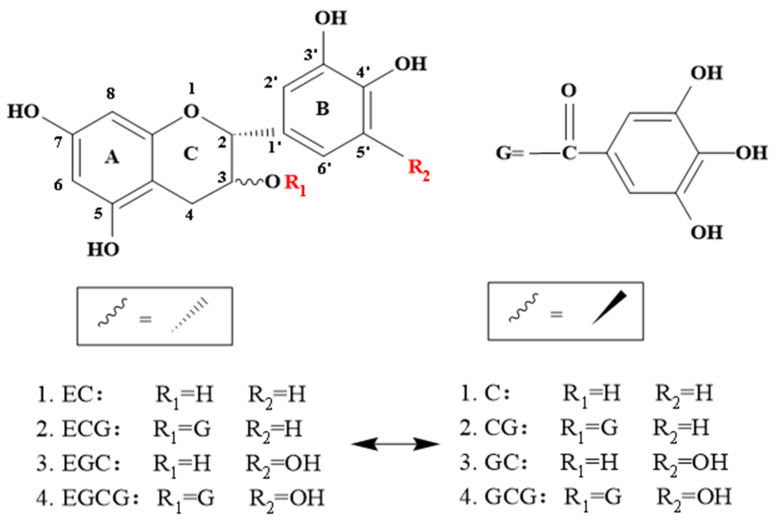
Chemical structure of eight catechins. EC, (−)-epicatechin; ECG, (−)-epicatechin gallate; EGC, (−)-epigallocatechin; EGCG, (−)-epigallocatechin gallate; C, (±)-catechin; CG, (−)-catechin gallate; GC, (−)-gallocatechin; GCG, (−)-gallocatechin gallate.

**Figure 2:**
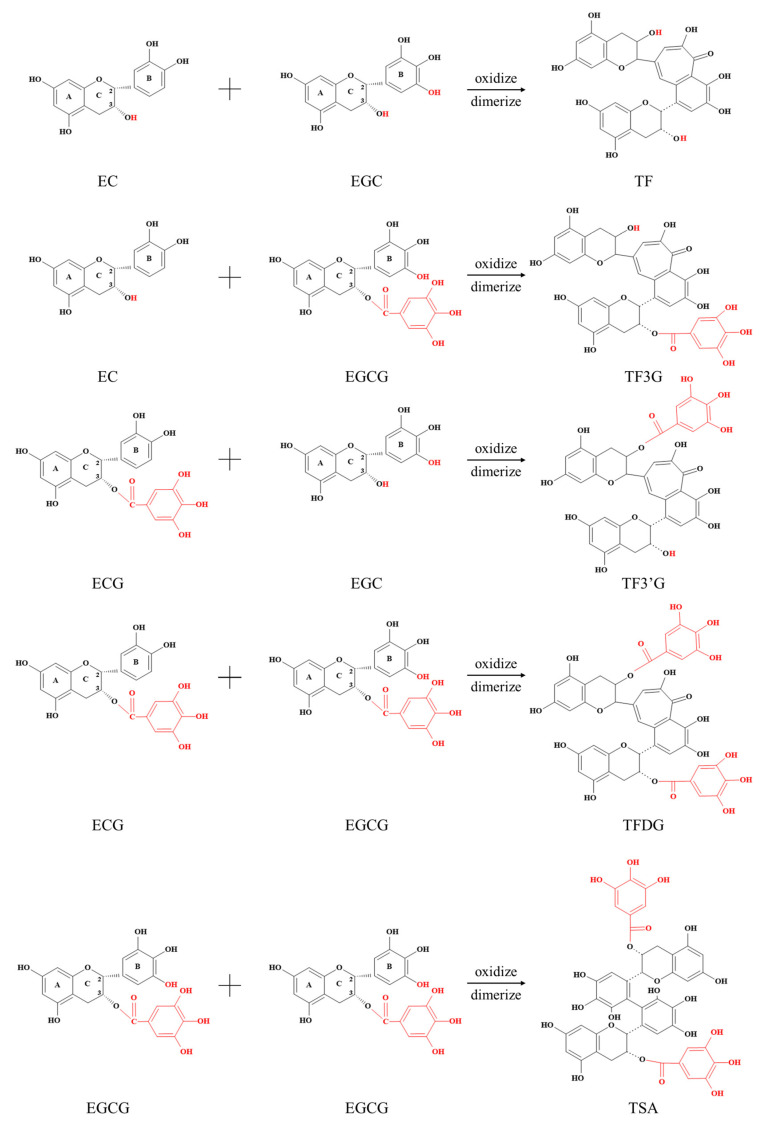
Oxidative dimerization reaction of catechins. TF, theaflavin; TF3G, theaflavin-3-gallate; TF3′G, theaflavin-3′-gallate; TFDG, Theaflavin-3,3′-digallate; TSA, theasinensin A.

The antioxidant activities of catechins are closely related to their structure, including hydroxyl groups at positions 5 and 7 of the A-ring, an ortho-3′4′-dihydroxyl group (catechol) or 3′4′5′-trihydroxyl group (pyrogallol) in the B-ring, and a gallate group located at position 3 of the C-ring (Figure 1) [13]. Structure (quantity and location) could also influence the activity of dimers. Catechins and dimers have regular differences in structure and are natural materials for studying structure–activity relationships. The effective antioxidant active groups in catechins and dimers can be screened through a clever comparison. Catechins are mainly composed of (−)-epigallocatechin gallate (EGCG), (−)-gallocatechin gallate (GCG), (−)-epicatechin gallate (ECG), (−)-catechin gallate (CG), (−)-epigallocatechin (EGC), (−)-gallocatechin (GC), (−)-epicatechin (EC), and (±)-catechin (C) [12]. Catechin was mainly dimerized through benzoquinone and a disproportionation reaction. Among them, the benzoquinone pathway refers to the oxidative polymerization of pyrogallol-type and catechol-type catechins to form TFs [14]. The disproportionation pathway refers to coupling oxidation between pyrogallol-type catechins to form TSs (Figure 2). Four main compounds of TFs are theaflavin (TF), theaflavin-3-gallate (TF-3-G), theaflavin-3′-gallate (TF-3′-G), and Theaflavin-3,3′-digallate (TFDG) [15]. There are many studies about the antioxidant activity of TFs, while that of theasinensin A (TSA) is little, especially in the comparison between TSA and TFs. Due to TSs and TFs being formed competitively during tea processing, it is worth studying which catechin dimer (TSA or TFs) has a stronger antioxidant activity and what their structure–activity relationship is. This is conducive to the targeted regulation of tea processing conditions as it enables us to obtain more dimers with strong antioxidant activities during production.

Based on these, this study systematically compared and analyzed the antioxidant activities of catechins and their polymers (CTOPs) through three antioxidant methods, exposed the relationship between the degree of oxidative polymerization and antioxidant activity, explored the antioxidant structure–activity relationship by using catechins and dimers with regular differences in structure, and disclosed the effects of the oxidative dimerization reaction on antioxidant activities of a substrate–catechin mixture. Our aim is to find the antioxidant active groups and analyze the effects of the degree of oxidative polymerization and oxidative dimerization reaction on the activity of catechins and polymers to enrich the cognition of the antioxidant activity of tea.

## 2. Materials and Methods

### 2.1. Chemicals and Reagents

A total antioxidant capacity assay kit with a ferric-reducing ability of plasma (FRAP) method (S0116) was purchased from Beyotime (Shanghai, China). 2,2′-Azinobis (3-ethylbenzothiazoline-6-sulfonic acid) (ABTS) was purchased from Meryer (Shanghai, China). 1,1-Diphenyl-2-picrylhydrazyl (DPPH) was purchased from Hefei Bomei Biotechnology Co., Ltd. (Hefei, China). Potassium persulfate was purchased from Macklin (Shanghai, China). Methanol was purchased from Merck KGaA (Darmstadt, Germany). 6-Hydroxy-2,5,7,8-tetramethylchroman-2-carboxylic acid (Trolox), C (HPLC ≥ 98%), GC (HPLC ≥ 98%), CG (HPLC ≥ 98%), GCG (HPLC ≥ 98%), EC (HPLC ≥ 98%), EGC (HPLC ≥ 98%), ECG (HPLC ≥ 98%), EGCG (HPLC ≥ 98%), TF (HPLC ≥ 95%), TF-3-G (HPLC ≥ 98%), TF-3′-G (HPLC ≥ 98%), and TFDG (HPLC ≥ 98%) were purchased from Yuanye (Shanghai, China). TSA (HPLC = 91.4%), TRs SII, and TBs were separated and prepared by us.

### 2.2. Comparison of Antioxidant Activities of CTOPs

A series of mass concentrations were prepared to compare the activities of samples with different degrees of oxidative polymerization. Samples, prepared before use, were dissolved in purified water and diluted to the required concentration.

#### 2.2.1. ABTS^+·^ Free Radical Scavenging Assay

This assay was carried out in line with the procedure as described previously [6], with mild adjustments. ABTS (7 mM) reacted with potassium persulfate (2.45 mM) in equal volumes for 12–16 h in the dark to prepare the ABTS^+·^ stock solution. ABTS^+·^ stock solution was then diluted with methanol to an absorbance of 0.70 (±0.02) at 734 nm, which was called the ABTS^+·^ reaction solution. The compounds were diluted to five different concentrations in 6.25 µg/mL~200 µg/mL. The ABTS^+·^ reaction solution (4 mL) was added to 100 µL of the compounds, and the blends were left for 10 min at room temperature in the dark. The absorbance was detected at 734 nm using a spectrophotometer (UV 3600, Shimadzu Corporation, Kyoto, Japan). Trolox and water, respectively, were served as the positive and negative controls. Taking ABTS^+·^ free radical scavenging activity (%, Equation (1)) as the ordinate and the mass concentration of the compounds as the abscissa, a linear regression equation was obtained, and the half maximal inhibitory concentration (IC_50_) of each compound was calculated.

ABTS^+·^ free radical scavenging activity (%) = (1 − OD_sample_/OD_NCK_) × 100 (1)

where OD_NCK_ and OD_sample_ are the absorbance of ultra-pure water and sample, respectively.

#### 2.2.2. DPPH Free Radical Scavenging Assay

This assay was carried out following the procedure reported previously [16], with slight modifications. In brief, 7 mg DPPH was dissolved in 100 mL of methanol to make a DPPH stock solution, and the compounds were diluted to five different concentrations in 50 µg/mL~800 µg/mL. The compound (10 µL) was reacted with 200 µL of a DPPH stock solution for 1 h at room temperature in the dark. The absorbance was detected at 515 nm using a Synergy H1 microplate reader (BioTek Instruments Inc., Winooski, VT, USA). Trolox and water were used as the positive and negative controls, respectively. Taking DPPH free radical scavenging activity (%, Equation (2)) as the ordinate and the mass concentration of the compounds as the abscissa, a linear regression equation was obtained, and the IC_50_ of each compound was calculated.

DPPH free radical scavenging activity (%) = (1 − OD_sample_/OD_NCK_) × 100 (2)

#### 2.2.3. Total Antioxidant Capacity Assay

Referring to the instructions, 180 μL FRAP reagent was reacted with 5 μL compound at 37 ℃ for 5 min, and the absorbance was detected at 593 nm using a Synergy H1 microplate reader. The mass concentrations of each sample were 1, 0.5, and 0.25 mg/mL. Blank control was water, while positive control was Trolox. The calibration curve was prepared using a FeSO_4_ standard solution (0.125 mM–4 mM). The ability to reduce iron ions was expressed as mM FeSO_4_ equivalent antioxidant capacity.

### 2.3. Structure–Activity Relationship of Catechins and Their Dimers in Antioxidant Activity

Catechins and dimers are natural materials for studying the structure–activity relationship due to their regular differences in structure. Effects of the structure of catechins and dimers on antioxidant activity were studied. 

This part mainly explored the effects of geometrical isomerism, B-ring structure, and the number of galloyl groups on the antioxidant activities of catechins. Meanwhile, the number and position of the galloyl group on the antioxidant activities of TFs were also studied. The antioxidant activity of TSA was compared with EGCG and TFDG. The molarities of each compound used in ABTS^+·^ free radical scavenging assay, DPPH free radical scavenging assay, and total antioxidant capacity assay were 100, 400, and 250 µM, respectively. The structural information of compounds is displayed in Table S1. The detection methods of antioxidant activity were similar to those in Section 2.2.

### 2.4. Influence of Oxidative Dimerization Reaction on the Antioxidant Activity of Catechins and Dimers

This study was carried out following our previous procedure [17]. Catechin dimeric oxidation products (product) are generated with the oxidative dimerization reaction of catechins (substrates). The antioxidant activity between dimers and related substrate monomers or substrate–catechin mixtures was compared next. It could help to learn whether the dimer or substrate–catechin held stronger antioxidant activity and explain the influence of the oxidative dimerization reaction on the antioxidant activity of substrate–catechin mixtures. The substrate–catechins corresponding to each dimer are shown in Figure 2. 

The molarities of each compound used in ABTS^+·^ free radical scavenging assay, DPPH free radical scavenging assay, and total antioxidant capacity assay were 100, 100, and 250 µM, respectively. The detection methods of antioxidant activity were similar to those in Section 2.2.

### 2.5. Statistical Analysis

All results were recorded as means ± standard deviations of at least three replicates. Comparisons between the two groups were performed with Student’s *t* test, and one-way analysis of variance with Duncan’s post hoc test was performed to measure the significant differences among multiple comparisons between compound effects. *p* < 0.05 and *p* < 0.01 were considered statistically significant.

## 3. Results

### 3.1. Comparison of Antioxidant Activities of CTOPs

This section systematically compared the antioxidant activity of CTOPs with three methods. Considering that the molecular weights of TRs, SII, and TBs were difficult to calculate, the activities of CTOPs were compared at a series of mass concentrations rather than molarities.

#### 3.1.1. ABTS^+·^ Free Radical Scavenging Activity

ROS are highly chemically reactive because they contain unpaired electrons. Free radical scavengers or antioxidants can provide electrons and inhibit oxidation. The ABTS^+·^ free radical scavenging assay and DPPH free radical scavenging assay indirectly reflect the antioxidant activity of compounds by detecting the ability of compounds to scavenge free radicals. Among them, the ABTS^+·^ free radical scavenging assay is fit for assessing the ability of compounds as hydrogen/electron donors, and for evaluating the antioxidant activity of compounds [18].

**Figure 3:**
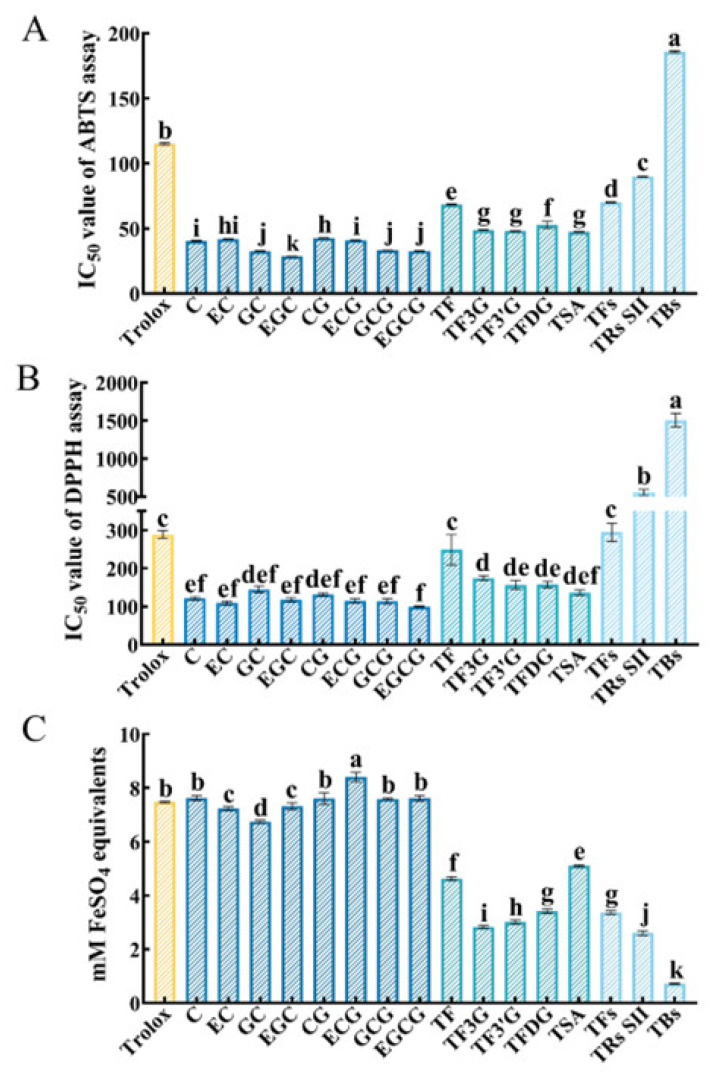
Antioxidant activities of catechins and their polymers (CTOPs) at mass concentrations. (**A**) ABTS^+·^ free radical scavenging activity of CTOPs; (**B**) DPPH free radical scavenging activity of CTOPs; (**C**) total antioxidant capacity of CTOPs. The concentration of samples used in total antioxidant capacity assay was 1 mg/mL. Theaflavins (TFs), thearubigins SII(TRs SII), and theabrownins (TBs) were components isolated from a tea sample using solvent extraction. ^a–k^ Different letters above the column indicate significant differences (*p* < 0.05).

Every sample dose-dependently scavenged the ABTS^+·^ free radical (Figure S1A—Supplementary Materials). IC_50_ of CTOPs were compared in Figure 3A. The ABTS^+·^ free radical scavenging activity of each sample was significantly stronger than Trolox (115 ± 1 µg/mL) except for TBs (186 ± 1 µg/mL), which showed that most of these samples obtained remarkable antioxidant potential. Tested samples could be classified into three categories according to their ABTS^+·^ free radical scavenging activity (*p* < 0.05): catechins, dimers, and polymers. From this view, the ABTS^+·^ free radical scavenging activity was negatively correlated with the degree of oxidative polymerization. At the same time, the ABTS^+·^ free radical scavenging activities of TFs (70 ± 1 µg/mL), TRs SII (90 ± 0 µg/mL) and TBs components isolated from a tea sample also manifested a higher degree of oxidative polymerization, the weaker the ABTS^+·^ free radical scavenging activity of these samples. 

#### 3.1.2. DPPH Free Radical Scavenging Activity

DPPH free radical is a neutral free radical with a single electron. When antioxidants are present, the DPPH free radical is eliminated. The scavenging mechanism of DPPH free radicals is mainly hydrogen atom transfer [19].

Similar to the results in the ABTS^+·^ free radical scavenging assay, every sample likewise scavenged the DPPH free radical in a dose-dependent manner (Figure S1B—Supplementary Materials). As shown in Figure 3B, the DPPH free radical scavenging activity of each sample was significantly stronger than Trolox (289 ± 11 µg/mL) except for TF (249 ± 40 µg/mL), TFs (295 ± 24 µg/mL), TRs SII (556 ± 41 µg/mL), and TBs (1505 ± 88 µg/mL). The DPPH free radical scavenging activities of catechins were stronger than those of dimers, followed by polymers. While that of TFs were significantly stronger than TRs SII, followed by TBs. These results signaled the capacity of CTOPs to clear away DPPH free radicals that were negatively related to the degree of oxidative polymerization. 

#### 3.1.3. Total Antioxidant Capacity

In the total antioxidant capacity assay with the FRAP method, ferric lessening ability was used to represent the total antioxidant capacity. Every sample reduced Fe^3+^-TPTZ in a dose-dependent manner (Figure S1C—Supplementary Materials). At the same concentration, the total antioxidant capacity of different samples was compared (Figure 3C). 

At the concentration of 1 mg/mL, the total antioxidant capacity of ECG (8.41 ± 0.17 mM FeSO_4_ equivalents) was significantly stronger than Trolox (7.48 ± 0.03 mM FeSO_4_ equivalents). No significant difference was discovered among GCG (7.59 ± 0.03 mM FeSO_4_ equivalents), EGCG (7.61 ± 0.1 mM FeSO_4_ equivalents), CG (7.61 ± 0.21 mM FeSO_4_ equivalents), C (7.62 ± 0.1 mM FeSO_4_ equivalents), and Trolox, while the total antioxidant capacities of other samples were significantly weaker than Trolox. Compared with the above two antioxidant indexes, the total antioxidant capacities of CTOPs were relatively lower (number of samples that obtained a stronger antioxidant activity than Trolox). The total antioxidant capacities of catechins were significantly stronger than those of dimers, followed by polymers. The total antioxidant capacities of TFs (3.37 ± 0.07 mM FeSO_4_ equivalents) were also significantly stronger than TRs SII (2.6 ± 0.09 mM FeSO_4_ equivalents), followed by TBs (0.72 ± 0.02 mM FeSO_4_ equivalents). These results implied the higher the degree of oxidative polymerization, the weaker the total antioxidant capacity of the samples. At a concentration of 0.5 mg/mL and 0.25 mg/mL (Figure S2—Supplymentary Materials), the total antioxidant capacities of dimers and polymers were also significantly weaker than Trolox, while most of the catechins did not obtain values significantly lower than Trolox. The total antioxidant capacities of catechins were significantly stronger than dimers, followed by polymers. The total antioxidant capacities of TFs were significantly stronger than TRs SII, followed by TBs. All these results were consistent with that at 1 mg/mL.

In conclusion, compared with Trolox, a commonly used positive control in the antioxidant assay, CTOPs showed stronger ABTS^+·^ free radical scavenging activities except for TBs as well as DPPH free radical scavenging activities except for TF, TFs, TRs SII, and TBs at mass concentration. The total antioxidant capacities of dimers and polymers were significantly weaker than Trolox, but most of the catechins were did not obtain values significantly lower than Trolox. Therefore, CTOPs possessed outstanding antioxidant activities to some extent. Studies have demonstrated that polyphenols are the main constituents of antioxidant activities in tea. The higher content of polyphenolic compounds existing in green teas made green tea extract show a more effective antioxidant activity [20]. Catechins have outstanding antioxidant activities [5] and the contents of catechins positively correlates with antioxidant activities [21]. Additionally, Chen et al. [8] found that catechin-oxidized polymers also have strong free radical scavenging activities, which was not much different from substrate–catechins. 

The results of different antioxidant methods were contradictory, mainly involving the comparison of different catechins or TFs. For example, the ABTS^+·^ free radical scavenging activity of TFDG was significantly weaker than TF-3′-G, while the total antioxidant capacity of TFDG was significantly stronger than TF-3′-G. There was no significant difference in the DPPH^·^ free radical scavenging activity between TFDG and TF-3′-G. ABTS^+·^ is a free radical with a positive charge. The ABTS^+·^ free radical scavenging assay detects the power of the ABTS^+·^ to abstract an electron or a hydrogen atom from the compound [5]. DPPH is a neutral free radical that could take in an electron of hydrogen radical to turn into a diamagnetic molecule [19]. Total antioxidant capacity assay with the FRAP method reflects the ferric-reducing ability of the sample, which mainly reflects the ability of electron transfer, one of the mechanisms of free radical scavenging. Therefore, the differences in the above results are derived from the diverse principles of these antioxidant methods. DPPH and ABTS^+·^ free radicals are chemical free radicals that are not naturally present in food or the human body and are far from the biological environment. Therefore, in addition to testing the ABTS^+·^free radical scavenging ability, DPPH free radical scavenging ability, and iron chelating ability of active ingredients, in future studies, we will also detect the antioxidant enzyme activity, ROS content, and oxidation product content in the body to verify the main conclusions obtained in this experiment.

Notably, although some small diversities in the results of three antioxidant methods were presented, a general trend in antioxidant activities could be concluded, i.e., when comparing the antioxidant activities of CTOPs at mass concentrations, the higher the degree of oxidative polymerization, the weaker the ABTS^+·^ free radical scavenging activity, DPPH free radical scavenging activity, and total antioxidant capacity of the samples. This was consistent with the results of Wang et al. [11] detected with the FRAP method. This study compared the antioxidant activity of catechins, dimers, and polymers at mass concentrations, which could help explain the antioxidant activities among different teas, such as green tea and black tea. The comparison among tea extracts (a mixture of many components) are usually carried out at the mass concentrations. Carloni et al. [5] tested the antioxidant activities of green, white, and black teas made of the same tea cultivar, and they found that the antioxidant activity of green tea was significantly stronger than black tea in the ABTS, ORAC, and LDL assays. As is widely known, green tea has more catechins than black tea because fermentation lessens catechin levels in the latter tea as catechins are converted to TFs and TRs. Therefore, the conclusion of Carloni et al. [5] was further demonstrated from the compound aspect in our results. Another study marked that at the same mass concentration, TFs isolated from black tea exhibited more antioxidant activities compared to TRs [22], which was also consistent with our results. Based on the above results and discussion, the decline of the antioxidant activity during black tea fermentation was at least partly due to the antioxidant activity of polymers being weaker than dimers, while that of dimers was weaker than catechins at mass concentrations (Figure S4).

In addition, the antioxidant activity seemed not to be simply influenced by the molarity of the sample. The molecular weight of EGCG is 458 g/mol, which is larger than EC (290 g/mol). At the same mass concentration, the molarity of EGCG is less than EC, but the antioxidant activity of EGCG was stronger than EC in every assay. Therefore, the higher antioxidant activity of EGCG could be caused by other reasons, such as the number and position of active groups. The same phenomenon was found in dimers. The antioxidant activity of TSA (914 g/mol), a compound with the highest molecular weight within the tested dimers, was significantly stronger than TF (565 g/mol) except for the total antioxidant capacity at 0.5 mg/mL. The influence of structure on the antioxidation activity of catechins and dimers will be studied in the following experiments.

### 3.2. Structure–Activity Relationship of Catechins in Antioxidant Activity

Catechins with a 2-phenyl benzo-pyran structure, belonging to flavanols, consist of three basic rings: A, B, and C [14]. The structural diversities of the 8 common catechins mainly exist in the B ring and C ring as shown in Figure 1. A pairwise comparison of catechins facilitated the discovery of the effects of structure on antioxidant activity.

#### 3.2.1. Screening of Antioxidant Active Group

**Figure 4:**
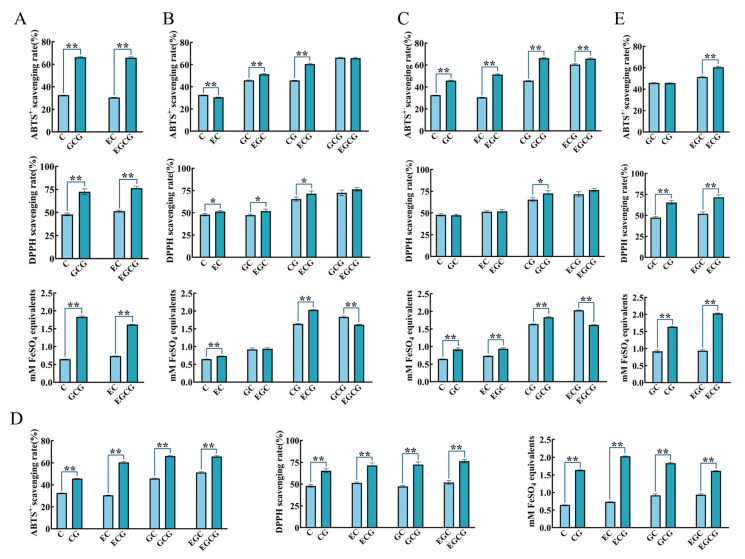
Structure–activity relationship of catechins with regard to antioxidant activities. (**A**) Screening of antioxidant active group. Effect of geometrical isomerism (**B**), B ring structure (**C**), and 3-galloyl groups (**D**) on antioxidant activities of catechins. (**E**) The dominant active group of catechins in antioxidant activity. ** *p* < 0.01. * *p* < 0.05.

The antioxidant activities between C and GCG or EC and EGCG were compared (Figure 4A); this was the comparison between catechins comprising a B-ring catechol but no 3-galloy and catechins simultaneously having a B-ring pyrogallol and 3-galloyl (Table S1). Results of the three antioxidant methods showed that the activity of GCG was significantly stronger than C, while that of EGCG was significantly stronger than EC, which proved that pyrogallol in the B-ring and 3-galloyl were possible antioxidant active groups of catechins.

#### 3.2.2. Influence of Geometrical Isomerism on Catechins’ Antioxidant Activity

Cis-catechins were compared with their corresponding trans-catechins to probe into the influence of geometrical isomerism on the activity of catechins (Figure 4B). In terms of the scavenging ABTS^+·^ free radical, C, EGC, and ECG were significantly stronger than EC, GC, and CG, respectively. There was no significant difference between GCG and EGCG. With regard to the scavenging DPPH free radicals, EC, EGC, and ECG were significantly stronger than C, GC, and CG, respectively. No significant differences was discovered between GCG and EGCG. The total antioxidant capacity of EC, ECG, and GCG was significantly stronger than C, CG, and EGCG, respectively. Moreover, GC and EGC had no significant differences between each other. To sum up, the comparison of antioxidant activities between cis-catechin and its corresponding trans-catechin had no accordant rule in different indexes. Therefore, geometrical isomerism was regarded as not an independent and critical factor affecting the antioxidant activities of catechins. Our results were confirmed by a previous report to a certain extent. Xu et al. [23] compared the antioxidant activity of tea epicatechins with their epimers through LDL oxidation, DPPH free radical assays, and a FRAP assay. They found that the majority of the noted diversities between epi-catechins and their corresponding epimers were tiny, even though they were occasionally statistically significant. Nevertheless, some studies offered different conclusions. Cis-catechins were more efficient in clearing away free radicals at high concentrations, while trans-catechins displayed stronger scavenging activities for macromolecular free radicals than cis-catechins at low concentrations [24,25,26]. Whether the above variant results are caused by discrepant sample concentrations and antioxidant models needs to be further verified.

#### 3.2.3. Influence of B Ring Structure on Catechins’ Antioxidant Activity

The phenolic hydroxyl group has a strong hydrogen-donating property, which can capture free radicals in the reaction system to achieve an antioxidant effect [27]. Both catechol and pyrogallol were disclosed as crucial substructures in heightening the antioxidant capacities of phenolic compounds [8]. Catechol and pyrogallol, which were stronger antioxidant substructures, will be studied in this experiment (Figure 4C).

In three indicators, the activity of GCG was significantly stronger than CG. The ABTS^+·^ free radical scavenging activity and total antioxidant capacity of GC and EGC were significantly stronger than C and EC, respectively. The ABTS^+·^ free radical scavenging activity of EGCG was significantly stronger than ECG, but the total antioxidant capacity of ECG was significantly stronger than EGCG. There were no significant differences between GC and C or EGC and EC or EGCG and ECG on the DPPH free radical scavenging activity. The above results hinted that pyrogallol was the stronger antioxidant substructure in the B-ring of catechins compared with catechol (except for the total antioxidant capacity of ECG and EGCG). This corresponded with the report of No et al. [28], which clearly showed that the pyrogallol in the catechin B-ring is the key structure for cleaning free radicals.

#### 3.2.4. Influence of 3-Galloyl Group on Catechins’ Antioxidant Activity

Catechins with a 3-galloyl group were compared with catechins without this group to confirm the antioxidant effect of 3-galloyl in catechins (Figure 4D).

In three indicators, the antioxidant activities of CG, ECG, GCG, and EGCG were significantly stronger than C, EC, GC, and EGC, respectively. These clear and coincident results adequately displayed that the 3-galloyl group heightened the antioxidant activity of catechins. This was consistent with a previous report, which indicated that the 3-galloyl group of ECG and GCG is the most vital structure for scavenging free radicals [28].

Based on the results of Section 3.2.2, Section 3.2.3 and Section 3.2.4, compared with other catechins, EGCG and GCG containing B-ring pyrogallol and 3-galloyl at the same time possessed stronger antioxidant activities at molarity, which corresponds in with the results in the literature [13,29].

#### 3.2.5. The Dominant Active Group of Catechins in Antioxidant Activity

As exposed in Section 3.2.3 and Section 3.2.4, B-ring pyrogallol and 3-galloyl were key antioxidant groups in catechins. An interesting question was whether B-ring pyrogallol or 3-galloyl had stronger antioxidant activities. To answer this question, ECG was compared with EGC, and CG was compared with GC (Figure 4E).

Results of the three antioxidant methods displayed that ECG had a significantly stronger activity than EGC. Additionally, the DPPH free radical scavenging activity and total antioxidant capacity of CG were significantly stronger than GC. No significant differences in ABTS^+·^ free radical scavenging activities were presented between CG and GC. The conclusion based on the above results was that 3-galloyl was a stronger antioxidant group in catechins than B-ring pyrogallol. Almajano et al. [29] reported that in the ABTS^+·^ radical scavenging assay, ORAC assay, and FRAP assay, the antioxidant activity of catechins was in the following order: ECG ≈ EGCG > EGC > EC (0.5 mM). The stronger antioxidant activity of ECG compared with EGC was consistent with our results.

### 3.3. Structure–Activity Relationship of Dimers in Antioxidant Activity

The structure–activity relationship obtained from catechin results was further verified by studying the influence of chemical structure on the activity of dimers. As dimers of catechins, TFs have a benzotropolone skeleton structure, while TSs possess a double flavanol skeleton structure. TFs and TSs contain different amounts of phenolic hydroxyl groups. In TFs, in addition to the original two phenolic hydroxyl groups on the A ring of each substrate–catechin, the structure formed by the B rings of two substrate–catechins through the benzoquinone pathway contains three hydroxyl groups. In TSs, in addition to the original two phenolic hydroxyl groups on the A ring of each substrate–catechin, the structure formed by the B rings of two substrate–catechins through the disproportionation pathway contains six phenolic hydroxyl groups [30]. The chemical structures of TFs and TSA are shown in Figure 2. 

#### 3.3.1. Influence of Number and Position of Galloyl Group on Antioxidant Activities of TFs

**Figure 5:**
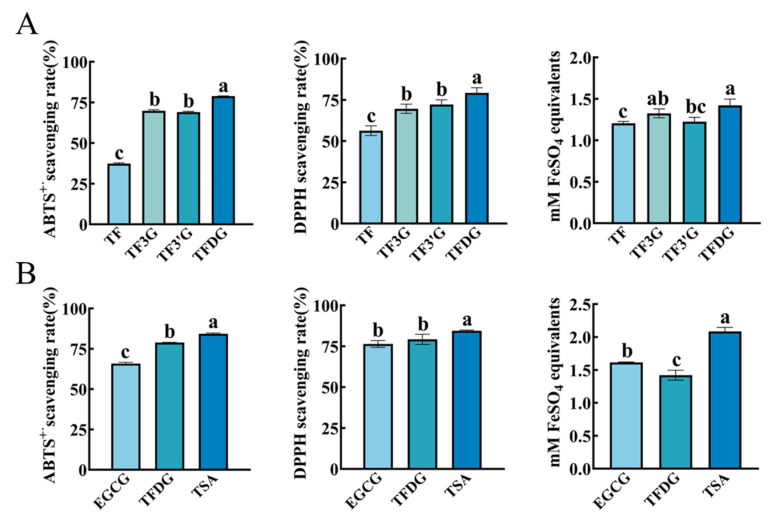
Structure–activity relationship of TFs and TSA with regard to antioxidant activities. (**A**) Antioxidant activities of TFs being influenced by the number but not the position of galloyl group; (**B**) antioxidant activity of TSA. ^a,b,c^ Different letters above the column indicate significant differences (*p* < 0.05).

The influence of the number and position of the galloyl group on the antioxidant activities of TFs was studied (Figure 5A). In terms of scavenging ABTS^+·^ and DPPH free radicals, TFDG showed a significantly stronger activity than TF-3′-G and TF-3-G, followed by TF. In terms of the ferric-reducing ability, TFDG had a significantly stronger activity than TF-3′-G and TF, while there were no significant differences between TFDG and TF-3-G or TF-3′-G and TF-3-G or TF-3′-G and TF. These results agreed with the results of the catechins in Section 3.2.4: the galloyl group was the vital antioxidant group and its number was positively correlated to this activity. Similar results have been reported in relation to the antioxidant activities of TFs (TF, TF-3-G, TF-3′-G, TFDG) strengthening when increasing the amount of gallate groups [19,31,32].

Leung et al. [31] reported that there is no difference in the inhibitory activity of Cu^2+^-mediated LDL oxidation between TF-3-G and TF-3’-G. Also, the position of the galloyl group did not affect the ABTS^+·^ free radical scavenging activity, DPPH free radical scavenging activity, and total antioxidant capacity in our results. Wu et al. [32] found that a superoxide radical, singlet oxygen (^1^O_2_), and H_2_O_2_ scavenging activity of TF-3′-G was stronger than that of TF-3-G, suggesting that the 3′-position gallate group in TFs may play a vital role in heightening their antioxidant activities. The reason for these differences in the above reports is unclear at present. One thing is for sure, TFDG, which simultaneously possesses 3- and 3′-galloyl groups, exhibited the strongest antioxidant activity in TFs.

#### 3.3.2. Antioxidant Activity of TSA Compared with EGCG and TFDG

TSA is the star compound of catechin dimers and has received widespread attention from researchers since its discovery. 

When compared at molarity, EGCG and TFDG were the representatives with the strongest antioxidant activity in catechins and TFs, respectively. It was shown that in three antioxidant methods, the activity of TSA was significantly stronger than TFDG and EGCG (Figure 5B). When considering the theory of structure, TSA possesses two galloyl groups and two pyrogallol groups (EGCG has one galloyl group and one pyrogallol group, while TFDG has two galloyl groups and no pyrogallol group), and the number of phenolic hydroxyl groups (16/molecule) is bigger than that of EGCG (8/molecule) and TFDG (13/molecule), which may result in a more prominent activity of TSA than EGCG and TFDG. Yoshino et al. [33] confirmed that TSs could chelate Fe^2+^ much stronger than EGCG, while O_2_^−^-scavenging activities of TSs were also better or nearly similar to that of EGCG. The results of the lipid peroxidation evaluation system showed that TSs had an excellent ability to inhibit lipid peroxidation compared with other polyphenols, and the effect was not much different from that of EGCG [34]. It is worth noting that the antioxidant activity of TSA was firstly compared with eight catechins and four TFs in this study, and TSA had the strongest antioxidant activity in all compounds (Figure S3—Supplementary Materials).

### 3.4. Influence of Oxidative Dimerization on the Antioxidant Activity of the Substrate Mixture

**Figure 6:**
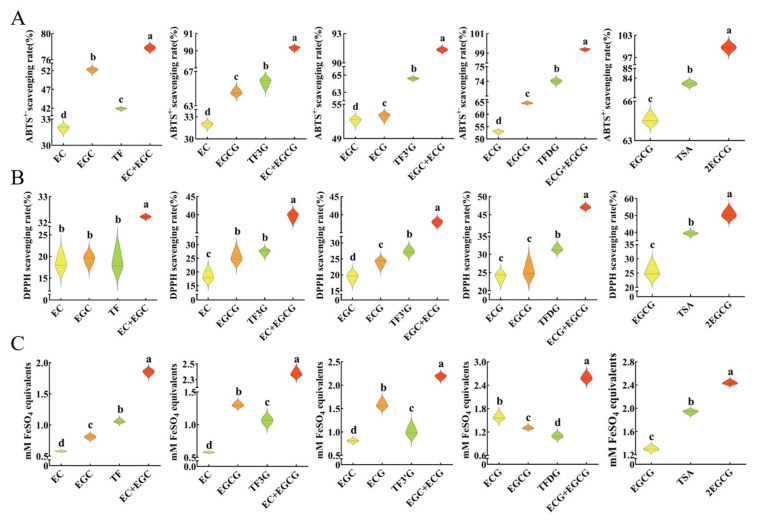
Effects of the oxidative dimerization reaction on the antioxidant activity of substrate–catechin mixture. The molarities of each compound used in ABTS^+·^ free radical scavenging assay (**A**), DPPH free radical scavenging assay (**B**), and total antioxidant capacity assay (**C**) were 100, 100, and 250 µM, respectively. ^a,b,c,d^ Different letters above the violin indicate significant differences (*p* < 0.05).

Firstly, the antioxidant activity between the product and related substrate monomer was compared (Figure 6). The ABTS^+·^ free radical scavenging activity of TF between EC and EGC was significantly different. There was no significant difference among TF, EC, and EGC in the DPPH free radical scavenging activity. The total antioxidant capacity of TF was significantly stronger than EC and EGC. The ABTS^+·^ free radical scavenging activity of TF-3-G was significantly stronger than EC and EGCG. The DPPH free radical scavenging activity of TF-3-G was significantly stronger than EC and was not significant difference in relation to EGCG. The total antioxidant capacity of TF-3-G between EC and EGCG was significantly different. The ABTS^+·^ and DPPH free radical scavenging activity of TF-3′-G was significantly stronger than EGC and ECG. The total antioxidant capacity of TF-3′-G between EGC and ECG was significantly different. The ABTS^+·^ and DPPH free radical scavenging activity of TFDG was significantly stronger than ECG and EGCG. However, the total antioxidant capacity of TFDG was significantly weaker than ECG and EGCG. The antioxidant activities of TSA in the three methods were significantly stronger than EGCG. The comparison of the antioxidant activity between product and substrate monomers varied in different indexes, but, mostly, the activity of dimers was not less than that of catechins. Jovanovic et al. [35] found that TF scavenged superoxide radicals at a higher rate than EGCG. Leung et al. [31] used Cu^2+^-mediated oxidation of human LDL as a model and confirmed that TFs have at least the same antioxidant capacities as catechins. Electroanalytical data revealed that TF had a stronger antioxidant potential and was a better copper chelator than EGCG after an interaction with copper [36]. There are also studies showing that EGCG has a stronger antioxidant capacity than TFs [37,38]. Hydrogen peroxide, hydroxyl radicals, peroxide anions, and superoxide anions are well-known reactive oxygen species (ROS). Lin et al. [37] reported that the superoxide scavenging abilities of theaflavins and EGCG are as follows: EGCG > TF-3-G > TF > TF-3,3′-G. However, in the same study, the restraint ability of xanthine oxidase activity was as follows: TF-3,3′-G > TF-3-G > EGCG > TF. Moreover, the order of H_2_O_2_ scavenging ability was TF-3-G > TF-3,3′-G > TF > EGCG, i.e., the antioxidant activity of dimers was not less than that of their individual substrate–catechin mixture. Leung et al. [31] also reported that in protecting human LDL from oxidation on the molar basis they gained the following ability: TF = EC > EGC, TF-3-G = EGCG > EC, ECG > TF-3′-G > EGC, TFDG > ECG, and TFDG > EGCG. Therefore, although the antioxidant activity of dimers was not more than any catechin, a stronger activity was found in dimers compared with their individual substrate–catechin mixture in the vast majority of experiments, which was consistent with our conclusion.

The antioxidant activity between the product and substrate mixture was then compared (Figure 6). It is interesting to note that the substrate mixture had a significantly stronger antioxidant activity than the product in all results. For example, the antioxidant activities of ECG + EGCG were significantly stronger than those of TFDG in three methods. Under the premise that the activity of dimer was not weaker than that of the related substrate monomer (in most cases), this result disclosed that the oxidative dimerization reaction hindered the antioxidant activity of the substrate–catechin mixture, which was another reason why the antioxidant activity during black tea fermentation declined (Figure S4).

Due to the presence of multiple hydroxyl groups in the structure of the galloyl group and pyrogallol, the number of galloyl groups, pyrogallols, and hydroxyl groups could be used to explain the differences between the activity of dimers and their substrates as well as the effects of the oxidative dimerization reaction on the antioxidant activity of catechins. TSA has twice as many galloyl groups, pyrogallols, and hydroxyl groups as its substrate EGCG; thus, TSA was significantly more active than EGCG at the same molarity. However, the activity of the one-molecule TSA was significantly weaker than that of the two-molecule EGCG. Hence, the antioxidant activity of the compound was not only affected by the number of its antioxidant active groups but could also be affected by the size of its molecular structure and the spatial location of its active groups. In addition, by analyzing the structure of TFs and their substrates, it was discovered that the number of galloyl groups in TFs is the sum of the two substrates, pyrogallol does not exist in TFs but lies in pyrogallol-type catechins which are the substrate of TFs, and the number of hydroxyl groups in TFs is greater than that of the substrate monomers but less than the sum of the two substrates. In short, the oxidative dimerization reaction weakens the antioxidant activity of the substrate–catechin mixture by reducing the number of active groups of the substrate and increasing the molecular structure size of the product. 

## 4. Conclusions

The effects of structures on the antioxidant activities of catechins and dimers was revealed, and the antioxidant active groups were screened in this study. Antioxidant activities of catechins were dominated by B-ring pyrogallol and 3-galloyl, but were not decided by geometrical isomerism. 3-galloyl was a stronger antioxidant group than B-ring pyrogallol in catechins. The number, not the position, of the galloyl group was positively correlated with the antioxidant activities of TFs. TSA has more antioxidant active groups (galloyl groups, pyrogallol groups, and phenolic hydroxyl groups) than EGCG and TFDG; thus, TSA had a stronger antioxidant activity. Additionally, this study found that the higher the degree of oxidation polymerization, the weaker the ABTS^+·^ free radical scavenging activity, DPPH free radical scavenging activity, and total antioxidant capacity of the samples. Under the premise that the antioxidant activities of dimers were greater than or equal to that of their substrate–catechin monomers (most of the time), the oxidative dimerization process significantly impaired the antioxidant activities of the substrate–catechin mixture (Table 1). Therefore, the degree of oxidative polymerization and oxidative dimerization reaction are not conducive to the antioxidant activity, which could reveal the mechanism of the descending antioxidant activity during the fermentation of black tea (Figure S4). Furthermore, the oxidative dimerization reaction weakened the antioxidant activity of the substrate–catechin mixture by reducing the number of active groups of the substrate and increasing the molecular structure size of the product. To sum up, the antioxidant active groups of catechins and dimers were screened and the effects of the degree of oxidative polymerization and oxidative dimerization reaction on their antioxidant activities was analyzed in this study, which could enrich the knowledge of the antioxidant activities of catechins and polymers. 

## Figures and Tables

**Table 1:** Effects of structure and oxidative polymerization on antioxidant activities of catechins, dimers, and polymers.

Indexes		Antioxidant Activity (DPPH, ABTS^+·^ and Total Antioxidant Capacity Assay in Non-Cellular System)
Structure–activity relationship of catechins	Geometrical isomerism	Not an independent interfering factor
	Catechol or pyrogallol in B-ring	Pyrogallol stronger than catechol
	3-galloyl group	3-Galloyl group stronger than the no-galloyl group
	Dominant active group	3-Galloyl group
Structure–activity relationship of dimers	Number of galloyl groups in TFs	Positively correlated with activities
	Position of galloyl groups in TFs	No influence
	Structure of TSA	Possessing strong activity at molarity due to having rich active groups
Oxidative polymerization	Dimers vs. substrate monomer	Dimers greater than or equal to the substrate monomer (in most cases)
	Dimers vs. substrate mixture	Dimers weaker than the substrate mixture (*p* < 0.05)
	Degree of oxidation polymerization (mass concentration)	Not positively correlated with the activity

## Data Availability

The data are contained within this article and the Supplementary Materials.

## Supplementary Materials

The following supporting information can be downloaded at: https://www.mdpi.com/article/10.3390/foods12234207/s1, Table S1: Catechins and TFs used in structure–activity relationship assay. Figure S1: Dose–effect relationship of antioxidant activities of catechins and their oxidized polymers (CTOPs); Figure S2: Total antioxidant capacity assay with FRAP method at 0.5 mg/mL and 0.25 mg/mL; Figure S3: Comparison of antioxidant activities of catechins and their dimers at molarity; Figure S4: Declining mechanism of antioxidant activity during black tea fermentation.
