# Supplementary material for: The Effects of Structure and Oxidative Polymerization on Antioxidant Activity of Catechins and Polymers
Source: Foods. 2023 Nov 22;12(23):4207. doi: 10.3390/foods12234207 (PMC10705921; doi:10.3390/foods12234207)
Supplement: Supplementary file 1 [file foods-12-04207-s001.zip › foods-2717890-supplementary.pdf]

Supplementary Materials

Table S1. Catechins and TFs used in structure-activity relationship assay

Compounds		Geometrical isomerism	B ring structure	Number of galloyl groups	Position of galloyl groups
Catechins	EGCG	cis-	pyrogallol	1	-
	ECG	cis-	catechol	1	-
	EGC	cis-	pyrogallol	0	-
	EC	cis-	catechol	0	-
	GCG	trans-	pyrogallol	1	-
	CG	trans-	catechol	1	-
	GC	trans-	pyrogallol	0	-
	C	trans-	catechol	0	-
TFs	TF	-	-	0	-
	TF-3-G	-	-	1	3
	TF-3′-G	-	-	1	3′
	TFDG	-	-	2	3 + 3′

Note: "-" means this column is not the object of study for this compound. EC, (−)-epicatechin; ECG, (−)-epicatechin gallate; EGC, (−)-epigallocatechin; EGCG, (−)-epigallocatechin gallate; C, (±)-catechin; CG, (−)-catechin gallate; GC, (−)-gallocatechin; GCG, (−)-gallocatechin gallate; TFs, theaflavins; TF, theaflavin; TF3G, theaflavin-3-gallate; TF3′G, theaflavin-3′-gallate; TFDG, Theaflavin-3,3′-digallate.

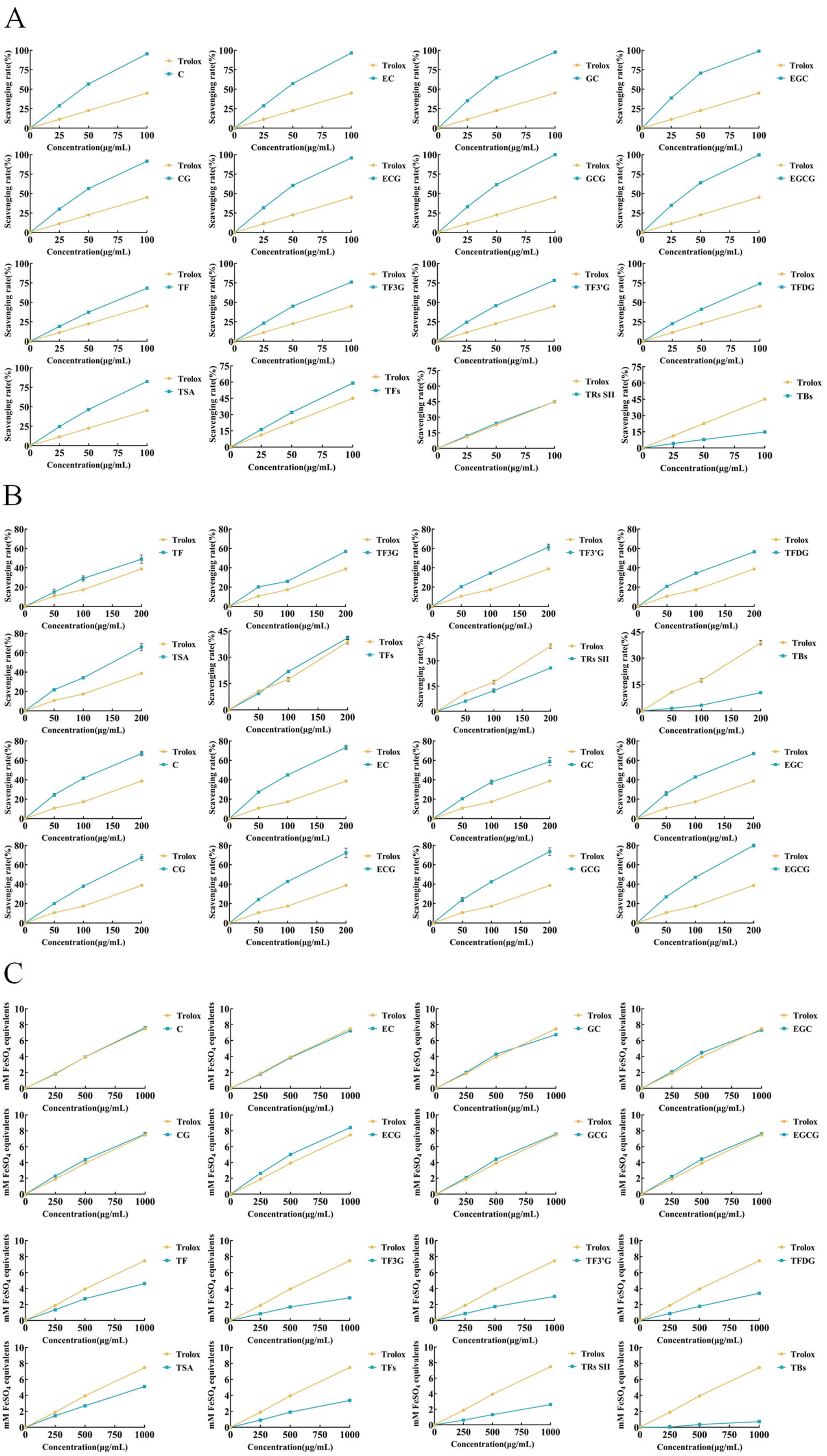
A
Scavenging rate(%)
Concentration(μg/mL)
Trolox
C
EC
GC
EGC
CG
ECG
GCG
EGCG
TF
TF3G
TF3'G
TFDG
TSA
TFs
TRs SII
TBs
B
Scavenging rate(%)
Concentration(μg/mL)
C
mM FeSO4 equivalents
Concentration(μg/mL)

Figure S1. Dose-effect relationship of antioxidant activity of catechins and their oxidized polymers (CTOPs). (A) Dose-effect relationship of ABTS^{+} free radical scavenging activity; (B) Dose-effect relationship of DPPH free radical scavenging activity; (C) Dose-effect relationship of total antioxidant capacity. Trolox was used as positive control. Theaflavins (TFs), thearubigins SⅡ (TRs SⅡ), and theabrownins (TBs) were components isolated from a tea sample by solvent extraction. TSA, theasinensin A.

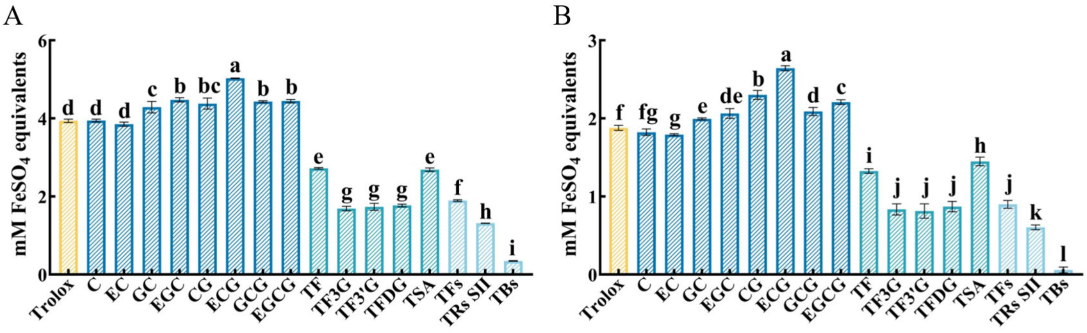

Figure S2. Total antioxidant capacity assay with FRAP method at 0.5 mg/mL (A) and 0.25 mg/mL (B).

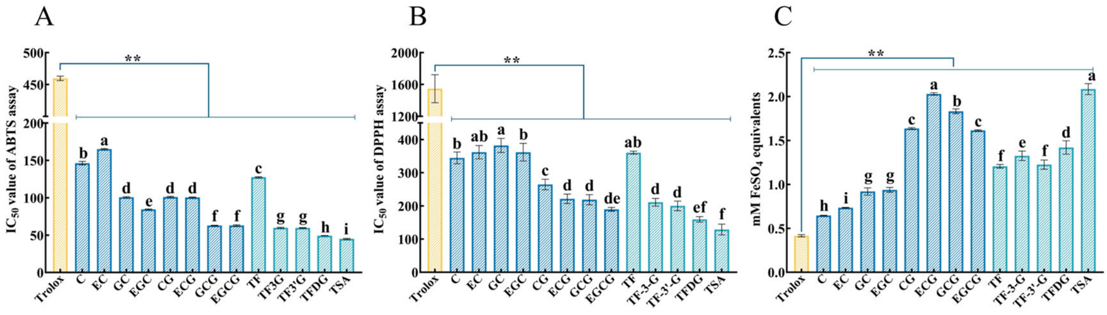

Figure S3. Comparison of antioxidant activities of catechins and their dimers at molarity. (A) ABTS$^{+\cdot}$ free radical scavenging activity of catechins and their dimers; (B) DPPH free radical scavenging activity of catechins and their dimers; (C) Total antioxidant capacity of catechins and their dimers. The molarity used in total antioxidant capacity assay was 250 μM. [a,b,c,d] Different letters above the column indicate significant differences ($p < 0.05$).

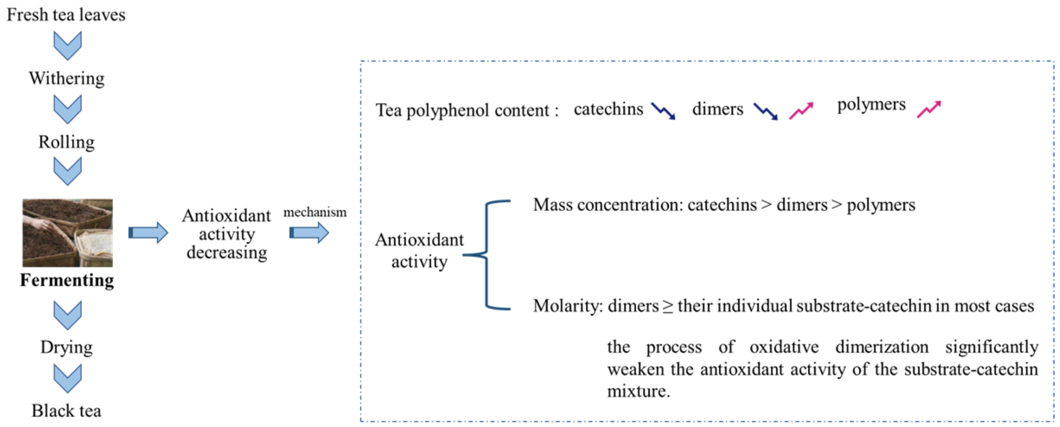

Figure S4. Declining mechanism of antioxidant activity during black tea fermentation.
